# Comparison of Maraging Steel Micro- and Nanostructure Produced Conventionally and by Laser Additive Manufacturing

**DOI:** 10.3390/ma10010008

**Published:** 2016-12-24

**Authors:** Eric A. Jägle, Zhendong Sheng, Philipp Kürnsteiner, Sörn Ocylok, Andreas Weisheit, Dierk Raabe

**Affiliations:** 1 Department Microstructure Physics and Alloy Design, Max-Planck-Institut für Eisenforschung GmbH, Max-Planck-Strasse 1, 40237 Düsseldorf, Germany; zhendong.sheng@iehk.rwth-aachen.de (Z.S.); p.kuernsteiner@mpie.de (P.K.); d.raabe@mpie.de (D.R.); 2 Institut für Eisenhüttenkunde, Rheinisch-Westfälische Technische Hochschule Aachen, Intzestrasse 1, 52072 Aachen, Germany; 3 Competence Area Additive Manufacturing and Functional Layers, Fraunhofer Institut für Lasertechnik, Steinbachstrasse 15, 52074 Aachen, Germany; soern.ocylok@ilt.fraunhofer.de (S.O.); andreas.weisheit@ilt.fraunhofer.de (A.W.)

**Keywords:** laser metal deposition, additive manufacturing, maraging steel, intrinsic heat treatment, precipitation strengthening, austenite reversion, atom probe tomography

## Abstract

Maraging steels are used to produce tools by Additive Manufacturing (AM) methods such as Laser Metal Deposition (LMD) and Selective Laser Melting (SLM). Although it is well established that dense parts can be produced by AM, the influence of the AM process on the microstructure—in particular the content of retained and reversed austenite as well as the nanostructure, especially the precipitate density and chemistry, are not yet explored. Here, we study these features using microhardness measurements, Optical Microscopy, Electron Backscatter Diffraction (EBSD), Energy Dispersive Spectroscopy (EDS), and Atom Probe Tomography (APT) in the as-produced state and during ageing heat treatment. We find that due to microsegregation, retained austenite exists in the as-LMD- and as-SLM-produced states but not in the conventionally-produced material. The hardness in the as-LMD-produced state is higher than in the conventionally and SLM-produced materials, however, not in the uppermost layers. By APT, it is confirmed that this is due to early stages of precipitation induced by the cyclic re-heating upon further deposition—i.e., the intrinsic heat treatment associated with LMD. In the peak-aged state, which is reached after a similar time in all materials, the hardness of SLM- and LMD-produced material is slightly lower than in conventionally-produced material due to the presence of retained austenite and reversed austenite formed during ageing.

## 1. Introduction

Maraging steels are materials that combine very high strength, hardness, and toughness [1]. Therefore, they are employed as tool steels in the mold and die making industry, but also for high-performance parts—e.g., in the aerospace industry [2]. They achieve their mechanical properties by a martensitic matrix that contains a high number density of nanometer-sized intermetallic precipitates [3,4,5,6]. Different from most tool steels, the martensitic microstructure is not achieved by a relatively high amount of carbon in the alloy composition, but instead by (usually) a high concentration of nickel. The almost complete lack of interstitial alloying elements leads to a good weldability of this class of alloys [7]. This, in turn, makes them amenable to metal additive manufacturing (AM) processes, in particular Laser Metal Deposition (LMD) and Selective Laser Melting (SLM) [8,9,10,11,12,13,14,15,16]. Since these processes involve a small melt pool generated by a laser beam for the consolidation of powder feedstock to a dense material, they share similarities with micro-welding processes.

One of the main strengths of AM processes is that very complex workpieces can be efficiently generated. In the toolmaking industry, metal AM processes are rapidly becoming the state of the art in the production of tool inserts for (polymer) injection molding processes. The geometrical freedom of AM allows to place cooling channels very close to the tool surface, yielding a very efficient cooling of the injected liquid polymer and avoiding ‘hot spots’ which would otherwise promote local material damage. Conventionally, cooling channels are produced by deep hole drilling which is only suited to produce (piecewise) straight cooling channels. Thus, cooling channels may not reach all locations in a complex tool and the fluid flow of coolant is hindered by turbulence induced by the rapid change of the channel axis where two bore holes intersect. It has been shown that, using AM-produced tool inserts, the heat removal from the tool can be enhanced such that the cycle time of the process is strongly reduced (by up to 60% [17]) and the productivity of the tool is equally improved.

Maraging steel that is used almost exclusively in AM processes today is the first-generation steel 18Ni-300, also known as ‘grade 300 maraging steel’ with the material number 1.2709 or slight modifications thereof, such as ‘Böhler V720^®^’ (material number 1.6354.9) [18,19]. Conventionally-produced (C-P) material is usually supplied in the solution annealed and quenched condition—i.e., fully martensitic without any precipitates present. These are formed during a subsequent ageing treatment, typically between 480 and 510 °C. Most AM processes used to synthesize metallic materials exhibit a rapid cooling rate during and after solidification (typically ~10^4^ K/s in LMD and up to 10^6^ K/s in SLM). It is therefore reasonable to assume that the microstructure of AM-produced (AM-P) maraging steel should also consist of martensite without precipitates. Indeed, it has been shown [20] that the microstructure of as-SLM-produced 18Ni-300 maraging steel does not contain any precipitates, however, it does contain a significant amount of retained austenite [13,20]. Normally, AM-produced parts made of maraging steel are not subjected to (thermo-)mechanical treatments—e.g., solution annealing or HIPing—before the final ageing, in contrast to hot-rolled C-P material. The microstructure of C-P and AM-P maraging steel at the start of the ageing treatment can therefore be expected to be quite different.

The aim of this paper is to investigate the difference in crystallography, chemical homogeneity on the micro- and nano-scale and phase distribution of C-P and AM-P 18Ni-300 maraging steel, and to determine the influence of these differences on the microstructural evolution during ageing treatment. For this purpose, we employ optical and electron microscopy including Electron Backscatter Diffraction (EBSD), Energy Dispersive X-ray Spectroscopy (EDS), as well as Atom-Probe Tomography (APT).

## 2. Materials and Methods

### 2.1. Additive Manufacturing

The C-P grade 300 maraging steel ‘Böhler V720^®^’ was produced by Böhler Edelstahl GmbH (Kapfenberg, Austria) via vacuum induction melting and vacuum arc re-melting. The material was received in form of a rolled bar and analyzed in the solution annealed (0.5 h at 820 °C) and quenched (rapid air quenching) condition. Its composition is given in Table 1.

LMD and SLM samples were produced using the parameters given in Table 2. Cuboids are produced by LMD using a simple unidirectional scanning strategy. The LMD samples were produced using a 3 kW diode laser. The powder is fed into the interaction zone of the laser beam and substrate via a coaxial powder feed nozzle. Argon is used as a carrier gas which also provides shielding from the surrounding atmosphere. The samples are 30 mm long (scanning direction, SD), 20 mm wide (transverse direction, TD), and 10 mm high, (build direction, BD). A detailed description of the production process of the SLM samples can be found in reference [13].

### 2.2. Microstructural Analysis

The AM-P samples were cut in two planes: one is parallel to the scan direction and the build direction (SD-BD) and the other is parallel to the transverse direction and the build direction (TD-BD). The C-P samples were cut parallel to the rolling direction. Standard metallographic sample preparation techniques, including a finishing step of polishing using colloidal silica suspension (OP-S from Stuers ApS, Ballerup, Denmark), were used. A solution of 1% HNO_3_ in ethanol was used to reveal the microstructure.

EBSD and EDS measurements were carried out in a JEOL 6500F (JEOL, Ltd., Tokyo, Japan) field emitter gun scanning electron microscope (FEG-SEM) equipped with an EDAX Octane Plus EDS detector and a TSL TexSEM DigiView EBSD camera using an acceleration voltage of 15 kV. TSL OIM Analysis™ software (version 7, EDAX, Mahwah, NJ, USA) was used for EBSD data analysis.

APT sample preparation was performed in a FEI Helios NanoLab 600i (FEI, Hillsboro, OR, USA) dual beam device employing the standard liftout process described in reference [21]. Tips were sharpened by applying annular milling patterns with a final step of low kV milling at 5 kV acceleration voltage to minimize Ga contamination at the surface. APT experiments were performed in a Cameca LEAP 3000 X HR (Cameca Instruments, Inc., Madison, WI, USA) in laser mode at a target temperature of 60 K, a laser energy of 0.4 nJ, and a laser pulse frequency of 250 kHz. The target evaporation rate was set to five atoms per 1000 pulses. Data analysis was performed using the IVAS software (version 3.6.6, Cameca Instruments, Inc., Madison, WI, USA).

## 3. Results

### 3.1. Ageing Behavior

**Figure 1:**
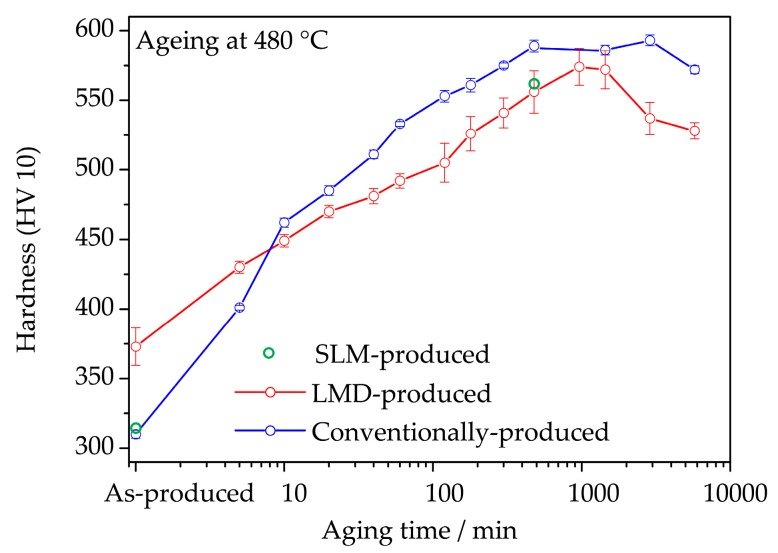
Microhardness of conventionally-produced and LMD-produced material as a function of ageing time at 480 °C. Additionally, the hardness of SLM-produced material in the as-produced state and after 480 min ageing is shown (data from [20]).

Figure 1 shows the microhardness of conventionally-produced (C-P), and LMD-produced (LMD-P) maraging steel samples as a function of ageing time at 480 °C. The values are an average of at least six hardness indents placed randomly in the middle of the sample (SD-BD plane) spaced at least 0.5 mm apart. Additionally, the microhardness of SLM-produced (SLM-P) material in the as-produced state and after 480 min ageing are shown (taken from reference [20]). Interestingly, the hardness of LMD-P material is higher than that of C-P and SLM-produced material in the as-produced and as-received states, respectively. The hardness of C-P and SLM-P material is roughly identical. This difference, however, vanishes quickly during ageing and, after 5 to 10 min of ageing, the LMD-P material becomes softer than the C-P material. Peak hardness is reached after 500 to 1000 min of ageing, and in this state the SLM-P material shows a similar hardness than the LMD-P material, both being about 50 HV softer than the C-P material. It is worth noting that the hardness drops after overageing occurs after a slightly longer time in the C-P material than in the LMD-P material. The origins of the differences in hardness, in particular the change in the hardness of the LMD-P material that shifts from being harder than C-P material (as-produced state) to being softer (peak aged state), will be investigated in detail in the remainder of the paper.

### 3.2. Microstructure in the As-Produced/As-Received State State

**Figure 2:**
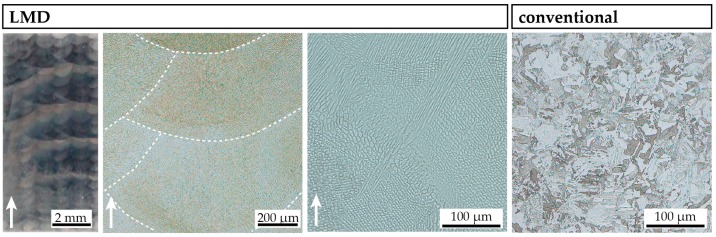
Optical micrograps of LMD-produced and conventionally-produced material in the as-produced/as-received state. Etching with HNO_3_ in ethanol reveals the melt pool boundaries and dendrites (LMD-produced material) and the martensite laths (conventionally-produced material). The white arrows indicate the build direction of the LMD-produced sample. LMD samples are cut in the TD-BD plane.

Optical micrographs of both C-P (right) and LMD-P material (left) in the as-produced/as-received state are shown in Figure 2. At low magnification, the layer-by-layer structure of the LMD-P material can be seen. The difference in contrast every four to five layers is an artifact from the etching. The build direction of the sample is upwards in the micrographs and the sample was cut in the TD-BD plane. At higher magnifications, the individual melt pools (delineated with dashed white lines in Figure 1), with many solidification dendrites within, are visible. They often cross melt pool boundaries, indicating epitaxial growth of grains between layers. At the highest magnification, it can be seen that the solidification structures are indeed dendrites, albeit with very short (secondary) side arms. The C-P material, on the other hand, does not show any of these solidification structures due to the thermomechanical processing it has experienced after primary synthesis. Instead, etching reveals a fine martensitic microstructure without preferred orientations of the martensite blocks. Note that no features typical for martensitic microstructures such as packets or blocks are visible in the LMD‑P material.

**Figure 3:**
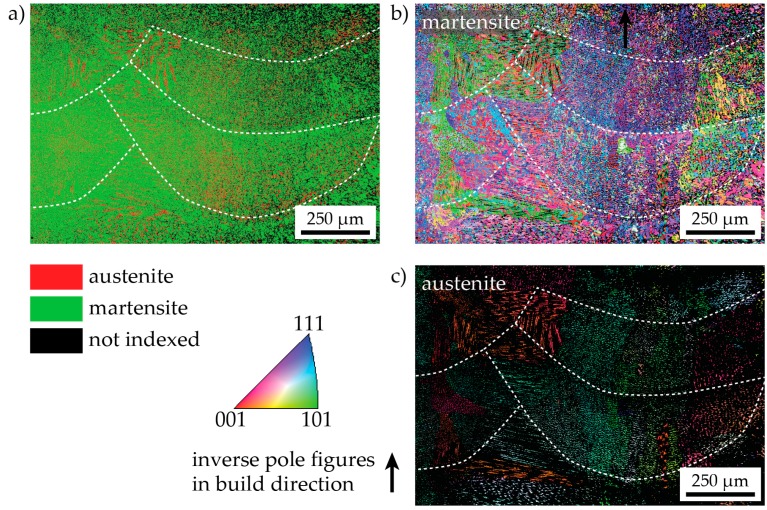
A large-area, low-magnification EBSD scan of as-LMD-produced material. (**a**) Phase map showing the location of the martensite and ferrite phases; (**b**) Inverse pole figure map of martensite only and (**c**) austenite only. Melt pool boundaries are indicated by dashed white lines. The black arrow indicates the build direction.

**Figure 4:**
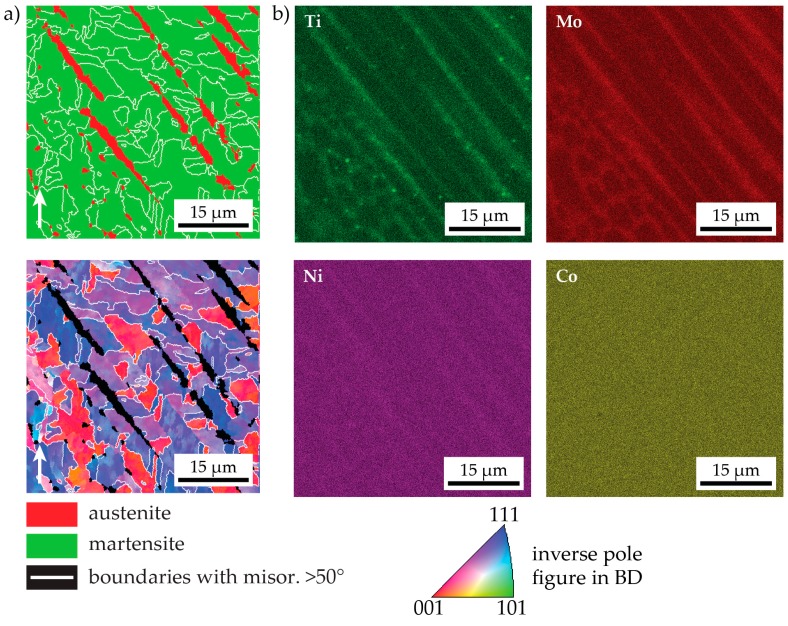
A small-area, high-magnification EBSD scan of as-LMD-produced material together with corresponding EDS element maps of various elements. In the phase map in panel (**a**), the location of the retained austenite in the interdendritic areas can be seen while in panels (**b**), the enrichment of Ti, Mo, and Ni in these regions is apparent.

The microstructure is investigated in more detail by EBSD and EDS in Figures 3, 4 and 6. Figure 3a shows the phases detected by EBSD over a relatively large part of the as-LMD-P sample (scan step size: 600 nm). Apparently, a considerable amount of retained austenite is present in the material. It is present in the entire sample, but not distributed entirely homogeneously. There is, for example, a lower apparent austenite fraction present in the areas just below melt pool boundaries. The austenite seems to be located along dendrite boundaries, but the magnification in this figure is not high enough to be certain. In panels (b) and (c), the crystallographic orientation of the martensite and retained austenite phases are shown, respectively. It can be seen that the austenite grains that formed upon solidification are quite large (up to one mm in diameter) and span several deposited layers. Due to this large (prior) austenite grain size and the resulting limited number of grains (and martensite variants) in the EBSD scan, it is not possible to make a statement about the overall crystallographic texture of the material.

**Figure 5:**
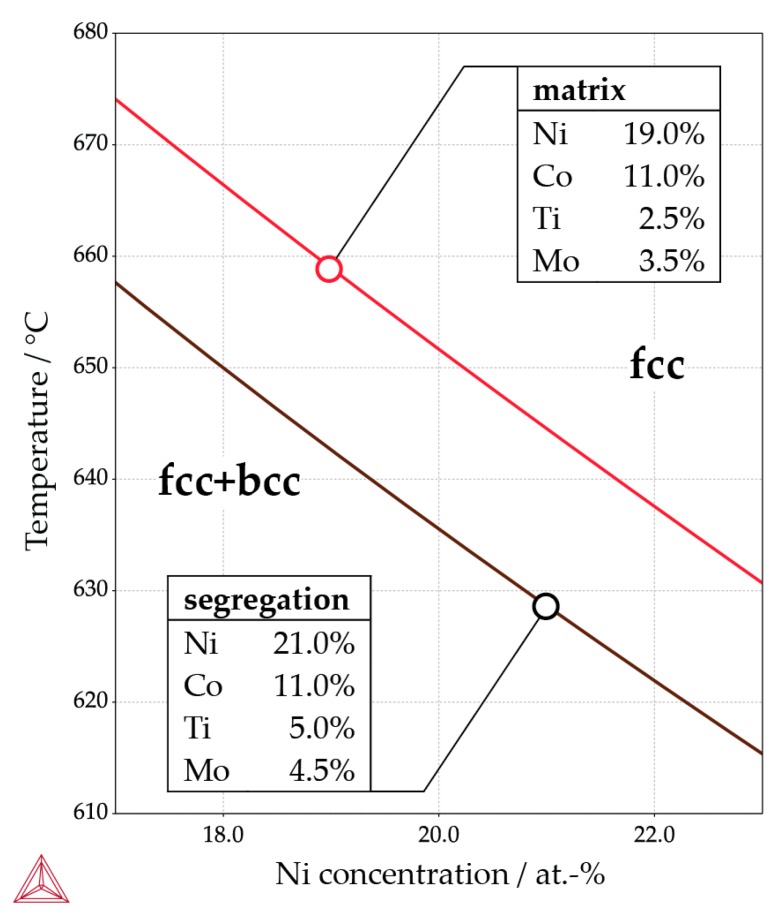
The relevant part of the phase diagram of the maraging steel as calculated by Thermocalc^®^. The lines separate the austenite and the austenite + ferrit phase fields. The results for two separate calucations are shown: one for the concentration of the interdendritic regions and one for the surrounding matrix. These concentrations were determined by EDS spot measurements. Austenite is stabilized by the solute segregation into the interdendritic regions.

The exact location of the retained austenite is displayed in Figure 4, where the phase map from a higher resolution EBSD scan (step size: 200 nm) is shown alongside corresponding EDS mappings of the Ti, Mo, Ni, and Co concentrations (the major alloying elements). The retained austenite indeed occurs in the interdendritic regions. Depending on the orientation of the dendrites, the austenite appears as a long needle in the EBSD phase map (when the dendrite axes are in the image plane) or as small circles (when the dendrite axes are perpendicular to the image plane). The interdendritic areas are enriched with Ti, Mo, and Ni. This is due to microsegregation during solidification—i.e., partitioning of solute elements into the remaining liquid during solidification. The enrichment in solutes explains why retained austenite is found only in these locations. Even though Ti and Mo are in general regarded as ferrite stabilizing elements, thermodynamic calculations show that in the interdendritic regions the austenite is stable to lower temperatures than in the matrix. This is depicted in Figure 5, where the relevant part of the phase diagram of the alloy is plotted as calculated by Thermocalc^®^ Version 2016a using database TCFE7 and not considering any phases besides austenite and ferrite in the energy minimization. The compositions of the matrix and interdendritic regions are determined by EDS spot measurements and are noted in the figure. Additionally, empirical equations for the calculation of the martensite start temperature, *M*_S_, of ultra-low carbon steels [22] predict that a higher content of Ti, Mo, and Ni all lower *M*_S_ and hence have an austenite-stabilizing effect.

In the EDS maps in Figure 4, also small (<1 μm) Ti-rich particles can be seen. They are most probably oxides formed due to the non-ideal shielding by inert gas during the process (cf. reference [23], wherein it was demonstrated that ODS-materials can be generated by LMD when the shielding gas is turned off).

It is difficult to quantify the exact amount of retained austenite in the LMD-P material. Due to the aforementioned large grain size and unknown crystallographic texture, X-ray diffraction measurements are unreliable. On the other hand, the austenite regions are so small (<5 μm in width) that high-resolution EBSD scans must be performed to correctly capture small austenite grains. Such high-resolution scans, however, only probe a small area and cannot reflect the slightly uneven distribution of austenite within the melt pools (cf. Figure 3). By averaging several small, high-resolution EBSD scans from the bottom to the top of the specimen, we estimate the volume fraction of retained austenite in the as-produced state as 8.5% ± 3.5%.

**Figure 6:**
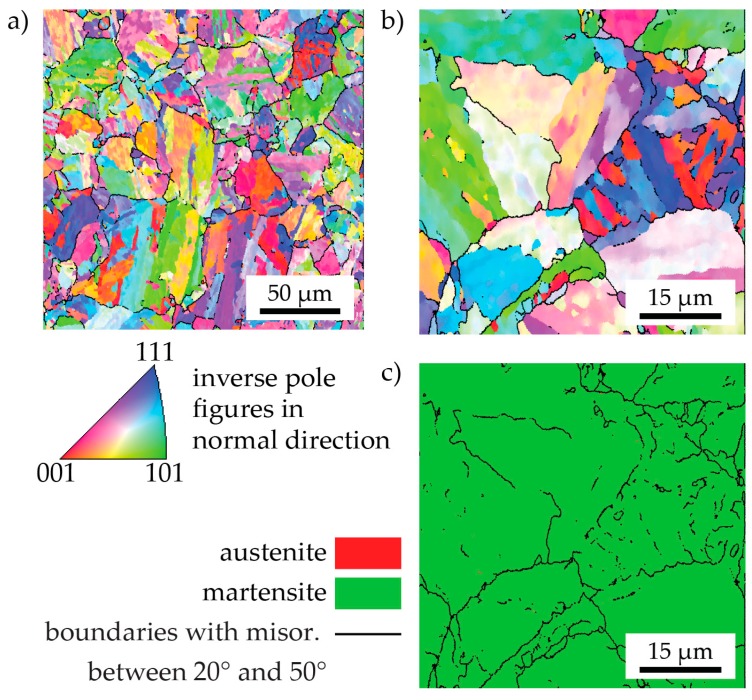
Two EBSD scans at low and high magnification of as-received, conventionally-produced material. Panels (**a**,**b**) show inverse pole figure maps while the phase map in panel (**c**) illustrates the absence of retained austenite.

The microstructure of as-received C-P material is shown in Figure 6. In the lower-resolution scan (step size: 500 nm) of panel (a), it can be seen that the crystallographic texture is random. The prior austenite grains (highlighted as boundaries in the martensite phase with a misorientation between 20° and 50°) are much smaller than in the LMD-P material (cf. Figure 3). A higher-resolution scan (step size: 200 nm, panels (b) and (c)) reveals that there is no detectable retained austenite in this material, in contrast to the LMD-P material. EDS measurements (not shown) confirm the chemical homogeneity of the material.

The appearance of the martensitic microstructure of the LMD-P material is very different from typical martensitic microstructures that contain a hierarchy of prior austenite grains, martensite packets, blocks, and laths. The martensite blocks (delineated by white lines in Figure 4, i.e., boundaries with misorientation above 50°) are in many places confined to a single dendrite, even though all dendrites belonging to the same prior austenite grain (i.e., all dendrites depicted in Figure 4) have only small mutual misorientation. The retained austenite acts as an additional spatial confinement for martensite blocks.

### 3.3. Hardness Drop in Topmost Layers of As-LMD-Produced Material

**Figure 7:**
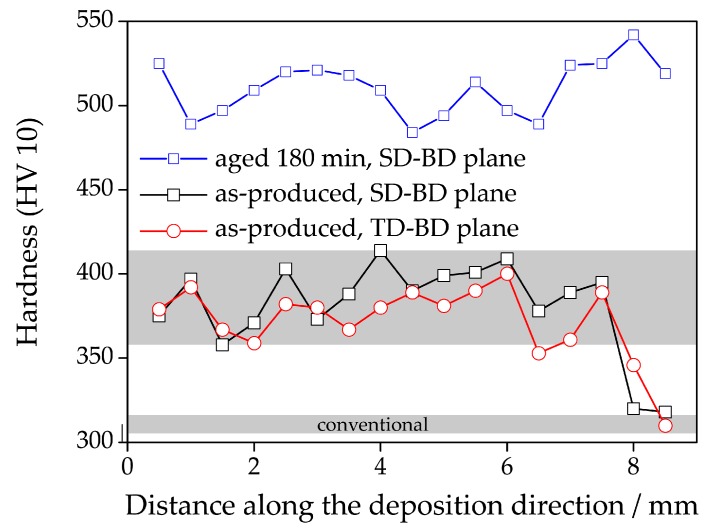
Microhardness of LMD-produced material as a function of distance from the substrate (build plate) along the build direction for as-produced material (on the build direction/scan direction surface as well as the build direction/transverse direction surface) as well as for material aged for 180 min (build direction/scan direction surface only).

Figure 7 shows a peculiar feature of the hardness of the as-LMD-P material. The hardness is constant along the build direction with the exception of the last few layers: At a height of ca. 7.5 mm above the base plate (2.5–3.5 mm below the sample surface), the hardness drops by about 70 HV to values of ca. 310 HV, comparable to the values of C-P and SLM-P material. Note that the scatter in the hardness values of LMD-P material is much higher than that observed for the C-P material (the range of hardness values of C-P materials is shown by the grey bar labelled ‘conventional’ in the figure). Yet, the observed hardness drop is clearly significant and reproducible. To check this one measurement, a series was performed on the plane build direction/scan direction, the other on the plane build direction/normal direction.

It is suspected that the higher hardness of LMD-P material compared to both C-P and SLM-P material in the as-produced/as-received state might be due to a difference in the nanostructure of the material—i.e., whether precipitates are present in the material or not. The fact that the upper layers of LMD-P material do not show this increased hardness supports this hypothesis. These are the layers that have experienced less intrinsic heat treatment in the process (i.e., less re-heating due to deposition of overlying layers). To investigate the hypothesis, APT measurements were performed. Liftouts were performed from an arbitrary position of C-P material, from the middle (in build direction) of both LMD and SLM-produced materials as well as from the topmost layer (middle of a scan track) of LMD-P material. No ageing heat treatment had been done on any of these specimens. The atom maps (not shown here) of all measurements do not show any remarkable features (see reference [20] for atom maps of SLM-produced material).

**Figure 8:**
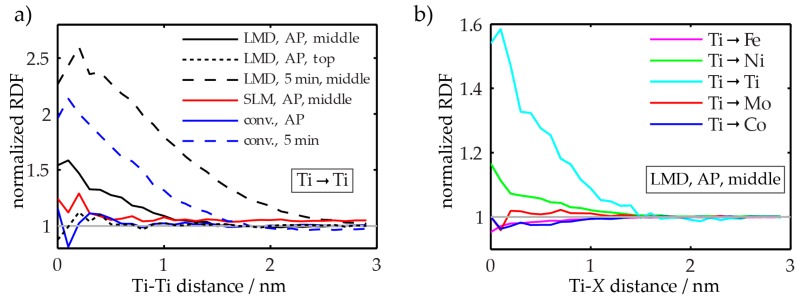
Radial distribution functions of various atom pairs normalized by their bulk average concentrations for different samples as a function of interatomic distance. (**a**) Ti-Ti RDFs for SLM- LMD- and conventionally produced samples in the as-produced (AP) state and after ageing for 5 min. Additionally, the RDF for a sample taken from the very top of as-LMD-produced material is shown; (**b**) Various RDFs (Ti as center atom and Fe, Ni, Ti, Mo, and Co as target atoms) of as-LMD-produced material (taken from the middle of the specimen). Panel (a) reprinted from [24] under the Creative Commons license (http://creativecommons.org/licenses/by/4.0/).

However, a statistical analysis of the atom positions reveals differences between the datasets. In Figure 8, radial distribution functions (RDFs), computed from these samples are shown. In panel (a), only the Ti-Ti RDFs of the various samples are plotted. The RDFs are normalized by the bulk (average) concentration such that a value of one for a given Ti-Ti-distance indicates that it is equally likely to find a Ti atom at this interatomic distance than it would be to find it in a random solid solution. For C-P and SLM-P material, the RDFs are equal to one (within the error of the measurement) for all Ti-Ti distances. However, for the LMD-P sample taken from the middle of the specimen, i.e., having experienced considerable intrinsic heat treatment, a value larger than one at small Ti-Ti distances is found. This indicates that it is more likely to find two Ti atoms close together than further away from each other, in other words, that clustering of Ti has begun. Note that this deviation from unity cannot be seen for LMD-P material taken from the very top of the specimen (see the black, dashed curve), i.e., for material that has not experienced significant intrinsic heat treatment.

### 3.4. Microstructure after Short Term (5 min) Ageing

**Figure 9:**
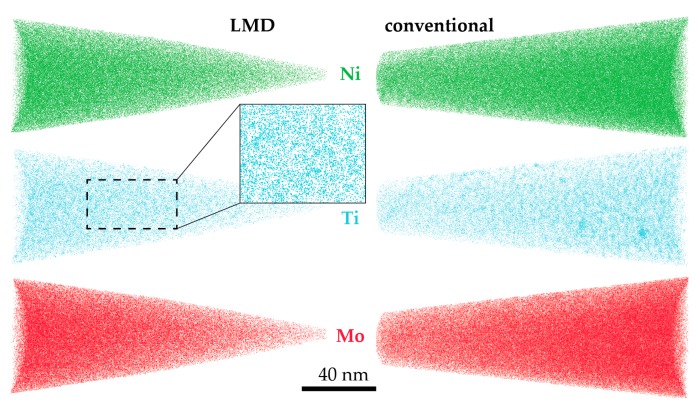
Atom maps obtained by APT of LMD-produced and conventionally-produced material after 5 min of ageing at 480 °C. In the Ti atom maps, early stages of precipitation are beginning to become visible.

After a short ageing treatment (5 min at 480 °C), both in LMD-P and in C-P material, strong Ti-Ti clustering occurs. This is indicated by the high values of the RDFs at low interatomic distances as determined by APT and as shown in Figure 8a. In panel (b), the type of clustering is shown: Apart from Ti-Ti RDF values being larger than one, also the Ti-Ni RDF is increased at small interatomic distances. This indicates that the clusters contain both Ti and Ni atoms and are possibly very small Ni_3_Ti precipitates, a phase that is expected to form in this steel. Corresponding atom maps for C-P and LMD-P material are depicted in Figure 9. Even though clustering is definitely detected in the statistical analysis and the hardness is already significantly increased compared to the as-produced state, the arrangement of the atoms visually still appears random with the possible exception of the Ti atoms (cf. the enlarged inset in the Ti atom map).

### 3.5. Microstructure in the Peak Aged (480 min) State

**Figure 10:**
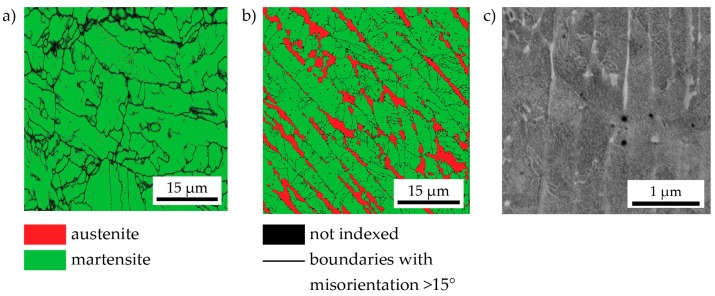
Small-area, high-magnification EBSD scans of conventionally-produced (**a**) and LMD-produced material (**b**) after ageing for 480 min at 480 °C. There is no reversed austenite in the conventionally-produced material while the increased fraction of austenite in the LMD-produced material as compared to the as-produced state indicates that austenite reversion has occurred; (**c**) an SEM micrograph of SLM-produced material aged at 480 °C for 480 min. Very fine austenite films along dendrite boundaries as well as fine precipitates are visible.

After ageing for 480 min—i.e., nearly in the peak-aged condition—the microstructure of the C-P maraging steel remains unchanged. In particular, still no austenite can be detected by a high-resolution EBSD scan (step size: 50 nm, see Figure 10a. The LMD-P material, on the other hand (scan step size: 200 nm), shows an increase in austenite fraction. It is now at 16.5% ± 3.5% as compared to 8.5% in the as-produced state (see, however, the discussion on the accuracy of this value in Section 3.2). This means that, in addition to the presence of retained austenite, reversed austenite is now also present in the microstructure. The same holds for aged SLM-P material (Figure 10c). Due to the very small dendrite width in this material, no reliable mapping of phases by EBSD was possible. Instead, an SEM micrograph of a lightly etched specimen is displayed that depicts very fine austenite films along dendrite boundaries as well as unidentified linear and spherical structures (possibly precipitates). The fcc (Cu type) crystal structure of the interdendritic films is confirmed in multiple spots by EBSD point measurements.

**Figure 11:**
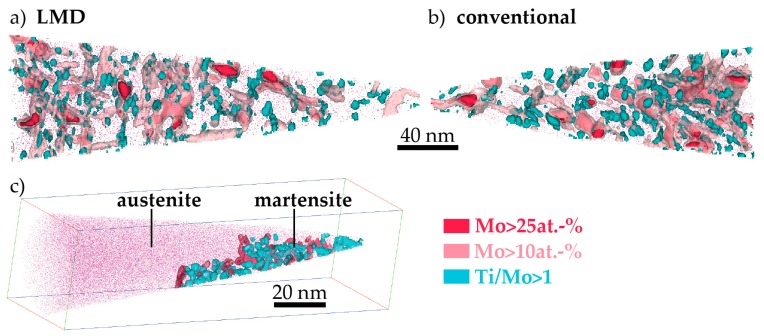
APT datasets of LMD-produced (**a**) and conventionally-produced material (**b**). Three different kinds of precipitates are present in both materials, as delineated by three different kinds of iso-concentration surfaces (panels (**a**,**b**) reprinted in modified form from [24] under the Creative Commons license (http://creativecommons.org/licenses/by/4.0/)). In panel (**c**), a measurement including both precipitate-containing martensite and precipitate-free austenite is shown.

In the nanostructure, as revealed by APT, a strong change has taken place upon ageing: A very high number density of precipitates emerges (see Figure 11). There are three different kinds of precipitates present: (Fe,Ni,Co)_3_(Ti,Mo), (Fe,Ni,Co)_3_(Mo,Ti), and (Fe,Ni,Co)_7_Mo_6_. The different precipitates are delineated in Figure 11 by three different iso-concentration surfaces: c(Mo) > 25 at %, c(Mo) > 10 at % and c(Ti) > c(Mo). In Table 3, the compositions and number densities of the various precipitates are compiled as determined by the constant concentration in the middle of precipitates found in proximity histograms based on the three previously mentioned iso-concentration surfaces. In our previous work [20], we analyzed SLM-produced material annealed for the same time and found the same kinds of precipitates. We speculated that the (Fe,Ni,Co)_3_(Ti,Mo)-precipitates were probably formed first, which we can now confirm by analyzing the APT measurements after 5 min annealing. Interestingly, the chemistry, number density, and sizes of precipitates in all three differently produced materials are very similar. Note that due to the interconnected nature of the (Fe,Ni,Co)_7_Mo_6_-precipitates, their number density cannot be determined. Due to the limited sample size and the chemical inhomogeneity of the material, only an approximation of the average number density values may be given.

An additional APT data set of LMD-P material in aged condition is displayed in panel (c) of Figure 11. In it, the two phases present in the maraging steel can be discerned: The martensite phase contains a high density of precipitates while the austenite phase is completely devoid of precipitates. The composition of the austenite is equal to the average composition of the alloy. Note that here, for LMD-P material, we did not find a Ni-enriched shell around the retained austenite indicative of austenite reversion as we did in the case of the SLM-produced material [20]. The EBSD measurements (cf. Figure 10), however, prove that austenite reversion does occur. The interface between austenite and martensite appears faceted, however the crystallographic orientation of the phases could not be determined from this particular APT measurement.

## 4. Discussion

The most striking differences between AM-produced and conventionally-produced 18Ni-300 maraging steel are summarized by the hardness versus ageing time curve (Figure 1). There are three notable effects:

(i). The hardness of as-LMD-produced material is higher than both as-SLM-produced and conventional as-received material. The reason for this lies in the early stages of precipitation that are detected by APT in the (and only in the) LMD-P material. Apparently, already the very small clusters have a significant strengthening effect. In principle, the precipitation (clustering) could occur either during the cooling down just after deposition and solidification of material in the LMD-process or during the pulse-like re-heating (intrinsic heat treatment) upon deposition of adjacent tracks and overlying layers. Both of these effects differ between SLM and LMD. In SLM, the melt pool is smaller and the scanning speed is higher than in LMD (cf. Table 2), leading to both a slower cooling rate after deposition and a less pronounced reheating during the intrinsic heat treatment. However, the results of Figure 7 allow to separate the effects of cooling rate and intrinsic heat treatment. Since the hardness of LMD-P material is comparable to C-P and SLM-P material in the very top layers, clearly the clustering in the as-produced state is due to the intrinsic heat treatment (that does not apply or applies less strongly to the very top layers). If the cooling after deposition were the origin of the clustering, it could also be observed in the top layers (cf. also the discussion in reference [24]). The fact that the intrinsic heat treatment is strong enough to induce (early stages of) precipitation in the present maraging steel, a material that needs several hours to reach a peak aged stage, suggests that the intrinsic heat treatment might be exploited to design a maraging steel that is fully in-situ precipitation strengthened—i.e., that does not need an additional heat treatment after AM production. We are currently optimizing the LMD-process and designing a model maraging steel to achieve this goal. First results with a Fe-Ni-Al alloy show promising results, including a very high number density of precipitates in the as-LMD-P state (publication in preparation).
(ii). The peak hardness is lower in both AM-produced materials compared to the C-P material. This is most likely due to the significant amount of comparatively soft retained (and reversed) austenite in the AM-produced materials (cf. Figure 3, Figure 4 and Figure 10). The austenite, in turn, is present because of the chemical inhomogeneity due to microsegregation during solidification. This microsegregation is present also in SLM-P material [13,20] that has been solidified at a higher rate than LMD-P material. Despite often being referred to as a process inducing rapid solidification, obviously neither LMD nor SLM enable effective trapping of solutes in the solidifying material in this particular alloy. Even though C-P material initially—i.e., after casting—surely also contained such inhomogeneity, these had been homogenized during the subsequent standard downstream thermomechanical processing such as hot rolling and annealing. Due to the near-net shape nature of AM processes this is not a viable option for AM-P material. Potentially, a prolonged solution annealing before ageing might remove the segregation and hence the retained austenite in AM-produced material, yet, it would also add an undesired additional processing step and additional cost to the AM production process. Note that the interdendritic spacing and thus also the width of the austenite regions is smaller in SLM-P material than in LMD-P material. Also the volume fraction of retained and reversed austenite—namely, 5.8% and 9.4% in the as-produced and peak aged states, respectively [13]—could be smaller, but this is not certain given the errors in the determination of the austenite content. The presence of reversed austenite in (over-)aged maraging steels is well documented (see e.g., [6,25,26]). Even though austenite lowers the strength of the material, it may be a desired microstructure constituent because it allows tailoring the ductility and toughness [27,28]. A slightly overaged condition may therefore be ideal for certain applications [29]. The exact influence of the fraction of retained and reversed austenite on the hardness of the materials is beyond the scope of this study due to the difficulty in separating the effects of grain size (prior austenite grain size, martensite block size, and morphology) and crystallographic texture from the austenite fraction.
(iii). The kinetics of precipitation is not noticeably different between C-P and AM-P material. It could have been expected that the presence of a high density of lattice defects in the AM-P material, originating due to the high residual stress imposed in the AM processes, leads to a quicker nucleation and growth of precipitates in the AM-produced materials or to a different morphology. This is, however, not the case. After 480 min of ageing, all differences in the nanostructure that have been present in the as-produced state are of no significance any more, as evidenced by the very similar composition, distribution, and sizes of the observed three types of precipitates (cf. Figure 11 and Table 3). Another point to note is a certain inhomogeneity in the LMD-P material. This is visible in the slightly uneven distribution of austenite and in the high scatter of the hardness values compared to the C-P material. We performed sets of microhardness indents with decreased load across a melt pool but did not find systematic changes in hardness. Hence, the observed variation in the hardness values seems to be truly random. A homogenizing heat treatment might alleviate this effect, too.

## 5. Conclusions

We studied the same nominal 18Ni-300 maraging steel alloy produced by three different processes: conventionally synthesized—i.e., by vacuum induction melting, vacuum arc re-melting, and hot rolling, Selective Laser Melting (SLM) and Laser Metal Deposition (LMD), two additive manufacturing methods. We find that the intrinsic heat treatment inherent to LMD (i.e., the heat input by adjacent tracks and overlying layers) is sufficient to induce early stages of precipitation in as-LMD-produced material, however, not in the topmost layers. This in turn leads to a higher hardness of as-LMD-produced material compared to as-received conventionally-produced material and as-SLM-produced material (except for the topmost layers). Upon ageing, however, the effect of the intrinsic heat treatment is superseded by the hardness increase due to precipitation. Precipitation kinetics and precipitate chemistry, size, and morphology is practically identical in all three studied materials. In the peak aged condition, the hardness of the two AM-produced materials is lower than that of the conventionally-produced material. This is due to the presence of both retained and reversed austenite in these materials, while there is no austenite at all in the conventionally-produced material. The reason for the austenite formation lies in the chemical inhomogeneity caused by microsegregation upon solidification that is suppressed neither in SLM nor in LMD, despite relatively quick cooling and solidification rates. This work illustrates that more microstructure and spatially resolving composition investigations need to be conducted to reveal and understand the micro- and nanostructures of allegedly well-known materials after synthesizing and processing them via the various novel AM methods. Additionally, our study demonstrates the possibility of designing materials that are in-situ precipitation hardened in the LMD process.

## Figures and Tables

**Table 1:** Chemical composition of the 1.6354.9 material used in this study determined by ICP-OES.

Alloying Element	C	Si	Mn	Mo	Ni	Al	Co	Ti	Fe
wt %	0.0018	0.025	0.011	5.03	18.3	0.077	8.74	0.68	Bal.
at %	0.0087	0.052	0.012	3.03	17.95	0.165	8.57	0.82	Bal.

**Table 2:** Processing parameters of two Laser Additive Manufacturing (LAM) methods for 18Ni maraging steels, Laser Metal Deposition (LMD), and Selective Laser Melting (SLM). The energy density is calculated by dividing the laser power by the scan speed, layer thickness, and laser focus diameter.

LAM Process	Laser Power (W)	Scan Speed (mm/s)	Laser Focus Diameter (μm)	Layer Thickness (μm)	Hatch Spacing (μm)	Energy Density (J/mm^3^)	Inert Atmosphere
LMD	800	10	1700	420	900	112.0	Ar
SLM Data from [13]	100	150	180	30	112	123.5	N_2_

**Table 3:** Chemical composition (in at %) of the precipitates in material produced by the three different processes (conventionally produced, C-P; Laser Metal Deposition-produced, LMD-P; and Selective Laser Melting-produced, SLM-P). The compositions and approximate number densities are determined from proximity histograms based on the three different iso-concentration surfaces (see text).

Precipitate	Material	Fe	Ni	Co	Mo	Ti	Number Density
		(at %)					(m^−3^)
(Fe,Ni,Co)_3_(Ti,Mo)	C-P	10	60	6	4	20	~4 × 10^23^
	LMD-P	15	61	2	3	20	~3 × 10^23^
	SLM-P [20]	12	60	4	5	18	–
(Fe,Ni,Co)_3_(Mo,Ti)	C-P	21	50	5	19	5	–
	LMD-P	22	52	2	22	2	–
	SLM-P [20]	22	52	5	16	5	–
(Fe,Ni,Co)_7_Mo_6_	C-P	40	17	4	39	0	~8 × 10^22^
	LMD-P	38	20	2	40	0	~5 × 10^22^
	SLM-P [20]	37	17	4	38	0	–

## References

[1] Sha W., Guo Z. (2009). Maraging Steels: Modelling of Microstructure, Properties and Applications.

[2] Kundig K.J.A., Myer K. (2002). Copper and copper alloys. Handbook of Materials Selection.

[3] Vasudevan V., Kim S., Wayman C. (1990). Precipitation reactions and strengthening behavior in 18 wt pct nickel maraging steels. Metall. Trans. A.

[4] Sha W., Cerezo A., Smith G.D.W. (1992). Atom Probe Studies of Early Stages of Precipitation in Maraging Steels. Scr. Mater..

[5] Tewari R., Mazumder S., Batra I.S., Dey G.K., Banerjee S. (2000). Precipitation in 18 wt% Ni maraging steel of grade 350. Acta Mater..

[6] Rao M.N. (2006). Progress in understanding the metallurgy of 18% nickel maraging steels. Int. J. Mater. Res..

[7] Lang F.H., Kenyon N. (1971). Welding of Maraging Steels.

[8] Stanford M., Kibble K., Lindop M., Mynors D., Durnall C. (2008). An investigation into fully melting a maraging steel using direct metal laser sintering (DMLS). Steel Res. Int..

[9] Cabeza M., Castro G., Merino P., Pena G., Román M. (2012). Laser surface melting: A suitable technique to repair damaged surfaces made in 14 Ni (200 grade) maraging steel. Surf. Coat. Technol..

[10] Grum J., Slabe J.M. (2007). The State of Differently Heat-Treated 12% Ni Maraging Steel after Laser Remelting. Mater. Sci. Forum.

[11] Thijs L., Humbeeck J., Bártolo P.J. (2011). Van Investigation on the inclusions in maraging steel produced by Selective Laser Melting. Innovative Developments in Virtual and Physical Prototyping.

[12] Yasa E., Kempen K., Kruth J. Microstructure and mechanical properties of Maraging Steel 300 after selective laser melting. Proceedings of the 21st International Solid Freeform Fabrication Symposium.

[13] Kempen K., Yasa E., Thijs L., Kruth J.-P., Van Humbeeck J. (2011). Microstructure and mechanical properties of Selective Laser Melted 18Ni-300 steel. Phys. Procedia.

[14] Becker T., Dimitrov D. (2016). The achievable mechanical properties of SLM produced Maraging Steel 300 components. Rapid Prototyp. J..

[15] Casalino G., Campanelli S.L., Contuzzi N., Ludovico A.D. (2015). Experimental investigation and statistical optimisation of the selective laser melting process of a maraging steel. Opt. Laser Technol..

[16] Casati R., Lemke J.N., Tuissi A., Vedani M. (2016). Aging behavior and mechanical performance of 18-Ni 300 steel processed by selective laser melting. Metals.

[17] LBC Engineering “Leistungsspektrum Lasergenerieren”. http://www.lbc-engineering.de/de/lasergenerierung.php.

[18] Decker R.F. (1979). Source Book on Maraging Steels.

[19] Decker R.F., Eash J.T., Goldman A.J. (1962). 18% Nickel maraging steel. Trans. ASM.

[20] Jägle E.A., Choi P., van Humbeeck J., Raabe D. (2014). Precipitation and austenite reversion behavior of a maraging steel produced by selective laser melting. J. Mater. Res..

[21] Larson D., Prosa T., Kelly T. (2013). Local Electrode Atom Probe Tomography—A User’s Guide.

[22] Liu C., Zhao Z., Northwood D.O., Liu Y. (2001). A new emperical formula for the calculation of ms temperatures in pure iron and super low carbon alloy steels. J. Mater. Process. Technol..

[23] Springer H., Baron C., Szczepaniak A., Jägle E.A., Wilms M.B., Weisheit A., Raabe D. (2016). Efficient additive manufacturing production of oxide- and nitride-dispersion-strengthened materials through atmospheric reactions in liquid metal deposition. Mater. Des..

[24] Jägle E.A., Sheng Z., Wu L., Lu L., Risse J., Weisheit A., Raabe D., Wu L., Risse J., Weisheit A. (2016). Precipitation Reactions in Age-Hardenable Alloys During Laser Additive Manufacturing. JOM.

[25] Shekhter A., Aaronson H., Miller M. (2004). Effect of aging and deformation on the microstructure and properties of Fe-Ni-Ti maraging steel. Metall. Mater. Trans. A.

[26] Galindo-Nava E.I., Rainforth W.M., Rivera-Díaz-del-Castillo P.E.J. (2016). Predicting microstructure and strength of maraging steels: Elemental optimisation. Acta Mater..

[27] Raabe D., Ponge D., Dmitrieva O., Sander B. (2009). Designing Ultrahigh Strength Steels with Good Ductility by Combining Transformation Induced Plasticity and Martensite Aging. Adv. Eng. Mater..

[28] Raabe D., Ponge D., Dmitrieva O., Sander B. (2009). Nanoprecipitate-hardened 1.5 GPa steels with unexpected high ductility. Scr. Mater..

[29] Viswanathan U.K., Dey G.K., Sethumadhavan V. (2005). Effects of austenite reversion during overageing on the mechanical properties of 18 Ni (350) maraging steel. Mater. Sci. Eng. A.

